# Genomic correlates of recombination rate and its variability across eight recombination maps in the western honey bee (*Apis mellifera* L.)

**DOI:** 10.1186/s12864-015-1281-2

**Published:** 2015-02-21

**Authors:** Caitlin R Ross, Dominick S DeFelice, Greg J Hunt, Kate E Ihle, Gro V Amdam, Olav Rueppell

**Affiliations:** Department of Computer Sciences, The University of North Carolina at Greensboro, Greensboro, NC 27402 USA; Department of Biology, 312 Eberhart Building, The University of North Carolina at Greensboro, 321 McIver Street, Greensboro, NC 27402 USA; Department of Entomology, Purdue University, West Lafayette, IN 47907 USA; School of Life Sciences, Arizona State University, Tempe, AZ 85287 USA; Department of Chemistry, Biotechnology and Food Science, Norwegian University of Life Sciences, 1432 Aas, Norway

**Keywords:** Meiotic recombination, Genome evolution, Red queen hypothesis, Hotspots, GC content, Sociality, Comparative genomics

## Abstract

**Background:**

Meiotic recombination has traditionally been explained based on the structural requirement to stabilize homologous chromosome pairs to ensure their proper meiotic segregation. Competing hypotheses seek to explain the emerging findings of significant heterogeneity in recombination rates within and between genomes, but intraspecific comparisons of genome-wide recombination patterns are rare. The honey bee (*Apis mellifera)* exhibits the highest rate of genomic recombination among multicellular animals with about five cross-over events per chromatid.

**Results:**

Here, we present a comparative analysis of recombination rates across eight genetic linkage maps of the honey bee genome to investigate which genomic sequence features are correlated with recombination rate and with its variation across the eight data sets, ranging in average marker spacing ranging from 1 Mbp to 120 kbp. Overall, we found that GC content explained best the variation in local recombination rate along chromosomes at the analyzed 100 kbp scale. In contrast, variation among the different maps was correlated to the abundance of microsatellites and several specific tri- and tetra-nucleotides.

**Conclusions:**

The combined evidence from eight medium-scale recombination maps of the honey bee genome suggests that recombination rate variation in this highly recombining genome might be due to the DNA configuration instead of distinct sequence motifs. However, more fine-scale analyses are needed. The empirical basis of eight differing genetic maps allowed for robust conclusions about the correlates of the local recombination rates and enabled the study of the relation between DNA features and variability in local recombination rates, which is particularly relevant in the honey bee genome with its exceptionally high recombination rate.

**Electronic supplementary material:**

The online version of this article (doi:10.1186/s12864-015-1281-2) contains supplementary material, which is available to authorized users.

## Background

In most organisms, sexual reproduction is linked to the formation of haploid gametes. The required reduction from two sets of homologous chromosomes to one recombined chromosome set per gamete occurs during the first meiotic cell division. Accordingly, the initiation of meiosis is characterized by the pairing of homologous chromosomes, which involves their physical connection and the induction of double strand breaks in the DNA. The subsequent DNA repair results in a sister chromatid exchange, a local gene conversion, or a recombination event (crossover) between homologous chromosomes [1]. One crossover per chromosome [2] or chromosome arm [3] are considered necessary for proper chromosome segregation, providing a minimum requirement for the number of crossovers [4]. Most species exhibit recombination rates close to this minimum requirement and recombination rates across a wide range of species are largely a function of physical genome size and chromosome number [5]. However, variation in recombination rates can be observed beyond this general pattern, as recombination rates vary substantially between species [3,6], within species [7], and within the same genome among and within chromosomes [8].

The western honey bee (*Apis mellifera*) exhibits an exceptional recombination rate and presumably presents the most notable deviation from the general rule that explains genome wide recombination rates in many other Eukaryotes. With twenty centi-Morgans per million base-pairs (20 cM/Mb), honey bees have the highest rate of recombination among Metazoans [9]. This high rate may be a genus-wide phenomenon [10]. The recombination rate varies substantially across the genome at the scale of one Mb [11], but it is independent of chromosome size [9]. Various explanations have been proposed, mostly accounting for the observation that other social species in the order Hymenoptera also seem to exhibit an elevated genomic recombination rate [9,12,13].

Two hypotheses that seek to explain the high recombination rates in the social Hymenoptera are based on the argument that recombination increases the genetic diversity in social insect colonies, which may increase the efficiency of division of labor among colony members or disease resistance [10,12-15]. However, theoretical studies show that the quantitative genetic variation in a colony is not significantly increased by recombination [16]. Another effect of increased recombination that may be beneficial is the reduced variation in genetic relatedness across multiple loci among colony members, which may reduce the potential for nepotism and kin conflict [17,18]. In addition, general reasons for elevated recombination rates that also apply to other taxa could explain the findings for social insects if the underlying selective forces are particularly strong in social insects: Relative small effective population sizes of social insects may promote the evolution of high recombination under selection, especially in haplo-diploid species where recessive alleles are expressed in the haploid sex [11]. Furthermore, social evolution may have exerted strong and antagonistic selection on a multitude of genes and the high recombination rates may be the result of divergent directional selection for the different female castes [19].

In contrast to these adaptive explanations, the high recombination rate might also be explained mechanistically. The GC content of the honey bee genome is only 33% on average with a strong bimodal distribution ranging from 10-70% [20] and genes are overly abundant in genome regions with lower than average GC content [21]. Thus, DNA structure and accessibility may be one mechanistic explanation [22]. Quantitative or qualitative differences in the recombination apparatus may also cause the overall excess of recombination in honey bees. However, little is known about the molecular mechanisms of recombination in honey bees and other social insects, despite a relatively good understanding of the process in other organisms [4]. Local, sequence-specific motifs also influence recombination rates [23] and several previous studies have analyzed the patterns of recombination in the honey bee genome at different scales to identify sequence motifs that drive honey bee recombination rate dynamics, as they do in several other species [24].

In selected 76 Mbp of the honey bee genome, recombination rate was found to be positively associated across 125 kbp windows with GC content, simple repeats, and the distance between genes, while negatively correlated to the proportion of low-complexity sequences [9]. The two smallest chromosomes were analyzed in more detail at the scale of about 30 kbp, and a select 300 kbp window at 3.6 kbp resolution, to reveal that true recombination hotspots are probably absent from the honey bee genome and that local variation in recombination rate is influenced by GC content and the three specific motifs CGCA, GCCGC and CCAAT [25]. At a yet finer scale (<1 kbp), the analysis of 444 recombination events between adjacent SNPs revealed three sequence motifs (CGCA, GCCGC, CCGCA) that were positively associated with recombination [26]. These studies used a powerful resolution but only a single data set for their analyses although recombination patterns vary intra-specifically [7] and theoretical reasons suggest recombination rate in honey bees may be even more variable than in other species [27].

To generate robust results from multiple data sets, we jointly analyzed genomic patterns of recombination rate from eight different, medium-density linkage maps of the honey bee and related recombination to genome sequence characteristics to contribute to a more conclusive understanding of local recombination rates and their variability in a genome with a globally elevated rate of meiotic recombination. The marker density in the different maps varied and allowed for an analysis at an intermediate scale, using 100000 base pair windows across the genome for our analyses. The analyzed maps were mostly derived from crosses of the North-American *Apis mellifera* population which represents an admixture of several ancestral populations [28] and overall similarities in the recombinational landscapes could not explained by ancestry alone [29]. We found that the local recombination rate was correlated to a large number of sequence features, which can best be explained by these features’ relation to GC content. Although the local recombination rates among maps were only moderately correlated [29], the results were largely consistent across maps. Furthermore, the variability of local recombination rates among maps correlated with the abundance of microsatellites and with some of the sequence motifs that correlated with the average recombination rate.

## Results

Based on eight medium-density linkage maps, we studied the relation between genome sequence features and local recombination rates. Overall, specific sequence characteristics were more strongly associated with local recombination rates than gene characteristics in our data set (Table 1). The correlation results were relatively consistent across the eight different maps, except for the “HBC” and the “R5” maps that revealed no major correlates of local recombination rate at all. Most of the DNA features that correlated positively with local recombination rate were also correlated with GC content. Independently, the abundance of microsatellites was positively linked to recombination rates. In the multi-factorial analysis, microsatellite abundance also correlated positively with the variance of recombination rate when the negative effect of the abundance of low complexity sequences was statistically accounted for (Table 1).

**Table 1:** All significant* bivariate correlation coefficients and standardized regression coefficients (in brackets) between sequence features and local recombination rate

**Feature:**	**Solig nac map**	**Grooming map**	**VSH map**	**JH map**	**HBC map**	**LBC map**	**R3 map**	**R5 map**	**Average recombination rate**	**Variance of recombination rate**
GC content	**0.11** (-0.18)	**0.25**	**0.30**	**0.20**		**0.14**	**0.13**		**0.27 (-0.29)**	
CpG	**0.14 (0.33)**	**0.27 (0.15)**	**0.33 (0.20)**	**0.23 (0.36)**		**0.17**	**0.16 (0.17)**	(0.07)	**0.32 (0.59)**	
GCCGC	**0.15**	**0.23**	**0.32** (0.14)	**0.22**		**0.19 (0.31)**	**0.12**		**0.30**	
CCGCA	**0.14**	**0.21**	**0.29**	**0.17** (-0.14)		**0.17**	**0.12**		**0.27**	
CCTCCCT	**0.14**	**0.25**	**0.29**	**0.20**		**0.10** (-0.13)	**0.11**		**0.27**	
CCAATCA	-0.05 (-0.06)									
CCCCGCAC	**0.09**	**0.14** (0.05)	**0.12**	0.06		**0.09**			**0.13**	
TGGGAAAGA			0.06	0.07					0.06	
Microsatellites	**0.08**	**0.13** (0.07)	**0.11**	**0.11**	0.09 (0.10)		**0.11**		**0.14** (0.05)	(0.07)
Low complexity	**-0.10**	**-0.23** (-0.11)	**-0.23**	**-0.19**		-0.09	**-0.09**		**-0.24**	(-0.06)
Gene number							**0.14 (0.15)**			
Gene size							**-0.10**			
Gene distance										
Intron size							**-0.10**			

*Significance at FDR < 0.05, FDR < 0.01 in bold for bivariate correlations; for multiple regression standard p < 0.05 is used and p < 0.01 shown in bold.

**Figure 1:**
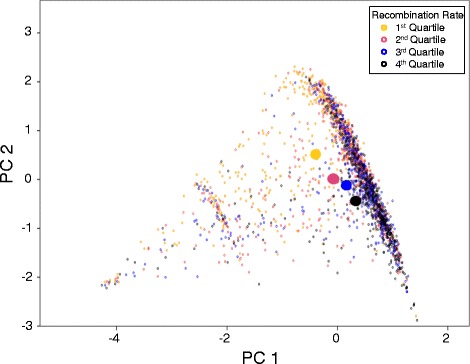
**Due to the high collinearity of the local abundance of the different di-, tri-, and tetra-nucleotide motifs, two principal components were extracted for each motif length that explained most of the variation in local recombination rates (see text).** For the analysis of di-nucleotides, shown here, both principal components differed significantly (p < 0.05) among genome intervals that exhibited low (first quartile), medium (second and third quartile), and high (forth quartile) average local recombination rates. Individual data are shown here as small circles and the 95% confidence interval or the group means as the larger solid ellipses. The principal component analysis of tri- and tetra-nucleotides revealed almost identical patterns.

Many di-, tri-, or tetra-nucleotide sequence motifs correlated significantly with local recombination rate. However, no single motif from these sets of correlated motifs emerged as a superior predictor of recombination rate. Instead, several motifs showed similar correlation coefficients to local recombination rate. In general, a motif’s correlation to recombination rate was influenced by its relative GC content, with high GC content motifs correlating positively to recombination rate. Most di-nucleotides showed a positive correlation to recombination rate in most maps. In contrast, the four di-nucleotides AA, AT, TA, and TT, were negatively correlated to recombination rate in most maps. The ratio of CG to GC, a specific indicator of DNA methylation [25], was positively correlated to recombination rate in most maps, although less so than CG or GC motifs on their own (Table 2). Two principal components (PCs) explaining a combined 96% of the variation were extracted from the di-nucleotide variables that were related to average recombination rate (Figure 1). The first PC was a positive predictor of average recombination rate (β = 0.19, p < 0.001). It was highly correlated to motifs with 50% and 100% GC content, the second PC was negatively associated with recombination rate (β = −0.27, p < 0.001). The second PC was positively correlated to di-nucleotide variables with 0% GC content and negatively correlated to variables with 100% GC content. The effects of these PCs were consistent between most maps with the exception of the “HBC” and “R5” data sets that yielded non-significant results. The second PC (β = −0.05, p = 0.022) but not the first (β = 0.02, p = 0.382) was also related to the variation of recombination rate among maps.

**Table 2:** Bivariate correlation coefficients between recombination rate and all di-nucleotide motifs that were significantly* correlated with local recombination rate in the eight different maps, its average and variance

**Motif:**	**Solignac map**	**Grooming map**	**VSH map**	**JH map**	**HBC map**	**LBC map**	**R3 map**	**R5 map**	**Average recombination rate**	**Variance of recombination rate**
AA	**-0.10**	**-0.10**	**-0.15**	**-0.10**	**---**	**---**	**---**	**---**	**-0.13**	**---**
AC	0.06	**0.17**	**0.19**	**0.12**	**---**	0.08	**0.10**	**---**	**0.17**	**---**
AG	**0.09**	**0.21**	**0.24**	**0.17**	**---**	**0.09**	**0.10**	**---**	**0.22**	**---**
AT	**-0.11**	**-0.16**	**-0.20**	**-0.15**	**---**	**-0.09**	-0.08	**---**	**-0.18**	**---**
CA	**---**	**---**	**---**	**---**	**---**	**---**	**---**	**---**	**---**	**---**
CC	**0.14**	**0.26**	**0.33**	**0.22**	**---**	**0.16**	**0.14**	**---**	**0.30**	**---**
CG	**0.14**	**0.27**	**0.33**	**0.23**	**---**	**0.17**	**0.16**	**---**	**0.32**	**---**
CT	**0.08**	**0.20**	**0.23**	**0.16**	**---**	**0.10**	**0.11**	**---**	**0.21**	**---**
GA	**0.09**	**0.24**	**0.26**	**0.19**	**---**	**0.11**	**0.11**	**---**	**0.24**	**---**
GC	**0.14**	**0.25**	**0.32**	**0.22**	**---**	**0.17**	**0.15**	**---**	**0.30**	**---**
GG	**0.14**	**0.27**	**0.33**	**0.23**	**---**	**0.16**	**0.14**	**---**	**0.31**	**---**
GT	0.06	**0.18**	**0.20**	**0.13**	**---**	**0.10**	**0.11**	**---**	**0.19**	**---**
TA	**-0.11**	**-0.18**	**-0.22**	**-0.17**	**---**	**-0.10**	-0.09	**---**	**-0.20**	**---**
TC	**0.08**	**0.23**	**0.26**	**0.19**	**---**	**0.12**	**0.12**	**---**	**0.24**	**---**
TG	**---**	**---**	**---**	**---**	**---**	**---**	**---**	**---**	**---**	**---**
TT	**-0.10**	**-0.10**	**-0.14**	**-0.10**	**---**	**---**	**---**	**---**	**-0.12**	**---**
CG/GC ratio	**0.09**	**0.23**	**0.20**	**0.17**	**---**	**0.10**	**0.11**	**---**	**0.23**	**---**

*Significance for FDR < 0.05; FDR < 0.01 is indicated in bold.

**Figure 2:**
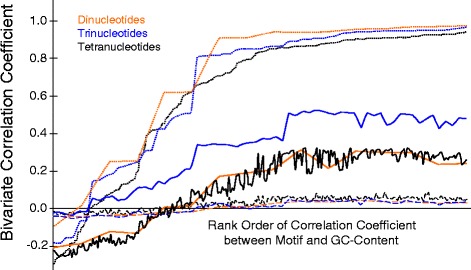
**Di-, tri-, and tetra-nucleotide motifs were rank-ordered along the x-axis according to their correlation with GC content (dotted lines).** Their correlation with the average local recombination rate followed a similar pattern (solid lines). This result suggests that GC content instead of specific nucleotide sequences is the most parsimonious explanation of local recombination rate in honey bees. Similar conclusions can be drawn for the variability of recombination rate (dashed lines).

The local recombination rate was correlated to numerous tri-nucleotide (Table 3) and tetra-nucleotide (Table 4) motifs. The most strongly correlated motifs were all positively associated with recombination rate. Many motifs exhibited very similar strengths of correlation in any given map and across maps, although the overall strength of correlation varied among maps, decreasing from the “VSH” and “Grooming” maps to the “HBC” and “R5” maps. No single tri- or tetra-nucleotide motif emerged as the main control of local recombination rate or its variance but some motifs were consistently correlated with recombination across data sets (Tables 3 and 4). These motifs were enriched for GC content (Figure 2). In contrast, the tri- and tetra-nucleotide motifs most negatively associated with recombination rate were extremely AT biased (“Grooming” map: TAA and AATA, “HBC” map: TGA and ATCA, “LBC” map: ATA and ACAT, “JH” map: TAA and AATA, “Solignac” map: AAT and TTAA, “VSH” map: TAA and TATT, “R3” map: TTA and TTAA, “R5” map: TCA and ATGA, recombination average: TAA and TTAA, and variance of recombination among maps: TAA and TTAA).

**Table 3:** Bivariate correlation coefficients between local recombination rate and the ten tri-nucleotide motifs that were most correlated* with the average local recombination rate

**Motif:**	**Solignac map**	**Grooming map**	**VSH map**	**JH map**	**HBC map**	**LBC map**	**R3 map**	**R5 map**	**Average recombination rate**	**Variance of recombination rate**
GCG	0.16 (2)	0.26 (12)	0.34 (1)	0.23 (9)	---	0.18 (3)	0.17 (2)	---	0.32 (1)	---
CGC	0.16 (1)	0.26 (14)	0.33 (6)	0.23 (8)	---	0.18 (4)	0.17 (1)	---	0.32 (2)	---
CGG	0.15 (3)	0.26 (13)	0.33 (2)	0.23 (10)	---	0.18 (2)	0.16 (7)	---	0.32 (3)	---
TCG	0.13 (17)	0.28 (1)	0.32 (14)	0.23 (5)	---	0.16 (7)	0.16 (3)	---	0.32 (4)	---
CCG	0.15 (4)	0.25 (17)	0.33 (4)	0.22 (14)	---	0.18 (1)	0.16 (8)	---	0.31 (5)	---
GAG	0.14 (10)	0.28 (2)	0.32 (10)	0.24 (1)	---	0.14 (20)	0.14 (18)	---	0.31 (6)	---
CGA	0.13 (20)	0.27 (3)	0.32 (15)	0.23 (4)	---	0.15 (11)	0.16 (5)	---	0.31 (7)	---
GGC	0.15 (5)	0.26 (16)	0.33 (5)	0.23 (6)	---	0.18 (6)	0.14 (16)	---	0.31 (8)	---
AGG	0.14 (9)	0.27 (10)	0.32 (11)	0.23 (3)	---	0.14 (23)	0.12 (23)	---	0.31 (9)	---
CTC	0.13 (16)	0.27 (4)	0.32 (9)	0.23 (2)	---	0.15 (12)	0.14 (14)	---	0.31 (10)	---

*Correlation coefficients are given when significant for FDR < 0.01, with relative rank of correlation strength in the individual analyses in brackets.

**Table 4:** Bivariate correlation coefficients between local recombination rate and the tetra-nucleotide motifs that were most correlated* with average local recombination rate

**Motif:**	**Solignac map**	**Grooming map**	**VSH map**	**JH map**	**HBC map**	**LBC map**	**R3 map**	**R5 map**	**Average recombination rate**	**Variance of recombination rate**
TCGG	0.15 (11)	0.27 (16)	0.34 (1)	0.23 (18)	---	0.17 (15	0.18 (1)	---	0.32 (1)	---
GGAG	0.15 (10)	0.28 (2)	0.33 (4)	0.24 (1)	---	0.14 (79)	0.13 (77)	---	0.32 (2)	---
CTCG	0.14 (46)	0.28 (1)	0.33 (13)	0.24 (6)	---	0.17 (23)	0.16 (20)	---	0.32 (3)	---
CGCG	0.16 (3)	0.25 (57)	0.33 (20)	0.23 (20)	---	0.18 (13)	0.16 (22)	---	0.32 (4)	---
GACG	0.15 (21)	0.28 (3)	0.32 (29)	0.23 (12)	---	0.16 (40)	0.17 (11)	---	0.32 (5)	---
GCGT	0.15 (24)	0.27 (11)	0.33 (6)	0.20 (60)	---	0.18 (11)	0.17 (16)	---	0.32 (6)	---
GCGC	0.17 (1)	0.25 (63)	0.32 (30)	0.23 (16)	---	0.19 (4)	016 (29)	---	0.32 (7)	---
TCGC	0.15 (14)	0.26 (26)	0.33 (19)	0.22 (33)	---	0.17 (27)	0.18 (3)	---	0.32 (8)	---
CGAG	0.14 (63)	0.28 (5)	0.33 (8)	0.24 (2)	---	0.16 (34)	0.16 (23)	---	0.32 (9)	---
AGCG	0.16 (4)	0.26 (38)	0.33 (5)	0.22 (53)	---	0.17 (22)	0.17 (15)	---	0.32 (10)	---

*Correlation coefficients are given when significant for FDR < 0.01, with relative rank of correlation strength in the individual analyses in brackets.

The first two PCs of the tri-nucleotide data set together explained 91.7% of the variation. The first PC was a positive predictor of average recombination rate (β = 0.22, p < 0.001) and was highly correlated to most motifs with at least one G or C nucleotide. The second PC was negatively associated with recombination rate (β = −0.25, p < 0.001) and was most positively correlated to pure AT motifs and negatively correlated to pure GC motifs. The first two PCs of the tetra-nucleotide data set explained 84% of the variation. The first of these PCs was a positive predictor of average recombination rate (β = 0.26, p < 0.001). The 50 most correlated variables with this PC consisted of 32 motifs with 50% GC content and 18 motifs with 75% GC content. The second PC was negatively associated with recombination rate (β = −0.21, p < 0.001). Among the top 50 correlated motifs to the second PC were 15 with 100% and 35 with 75% AT content, while all 16 possible 100% GC motifs were among the twenty most negatively correlated motifs.

## Discussion

We analyzed in parallel eight different, medium-density recombination maps of the honey bee genome at an intermediate scale. The maps represented a number of different honey bee populations and sources and varied significantly with regards to the similarity of their local recombination estimates [29]. Nevertheless, our results were quite consistent among maps, indicating that GC content may be the most important determinant of local recombination rates in the honey bee genome at the investigated scale. This finding corroborates the conclusions of a previous study of 125 kbp windows [9] but contrasts with results of a more recent study that was performed at different scales [25].

The two exceptional maps in our data set were the “R5” and “HBC” maps, which did not reveal any significant patterns. This discrepancy is difficult to explain because each of these maps had one closely related “sibling” map that were constructed with similar methods and derived from genetically related sources. Overall, the local recombination rates were more similar between these pairs (“R3” and “R5”, “HBC” and “LBC”) than between them and the other maps [29]. Yet, the “R3” and “LBC” map analysis yielded results that conformed to the findings from the remaining four maps, while “R5” and “HBC” map analysis did not. We cannot explain this phenomenon, but note that these four maps were constructed with relatively few markers. Thus, the recombination profiles are less detailed and relatively few changes may alter the overall results. The resolution of the different maps generally increased the correlation with specific DNA motifs and features, but the highest resolution map (Solignac) did not exhibit the highest correlation coefficients. We cannot exclude biological reasons due to the European origin of the “Solignac” map [11]. However, the phylogenetic distance between mapping populations was only a poor predictor of their overall similarity in local recombination rate [29] and a methodological explanation seems more likely. Both maps that were constructed solely based on chip-based SNP genotyping [30,31] showed relatively high correlations with the different variables despite their very different population origin. Presumably, the results indicate a superior accuracy of the maps that have been SNP-genotyped. Other studies indicate that SNP genotyping in general is more accurate than microsatellite genotyping [32,33]. Both SNP-based data sets in our study showed the highest correlation coefficients with the different variables.

Common results emerge from six of the eight investigated maps, and we can conclude that our findings are largely independent of the specific recombination pattern in each map and represent more general principles. Limited by the resolution of the maps, our analysis cannot be directly compared to comparative results in *Drosophila* that were performed at a much finer scale [7]. Nevertheless, our conclusions about correlates of the local recombination rates at the 100 kbp scale should be robust. In addition, our analysis allowed an assessment of whether specific DNA features are associated with variability in local recombination rates, a question that is particularly relevant in the honey bee genome with its exceptionally high recombination rate [27].

Our results confirmed previous findings that GC content is a major correlate of local recombination rate in honey bees [9,25]. This correlation may be due to biased gene conversion that enriches areas of high recombination for GC [34]. In contrast to *Drosophila* [7,35], biased gene conversion may be operational in honey bees [36], even though the honey bee genome is relatively AT-rich and experiences a high recombination rate [20]. In the honey bee genome, GC content may also cause higher local recombination rates because it is correlated to gene density [20] and thus accessibility of the DNA [37], which increases local recombination rates in other organisms [38]. However, gene density itself did not emerge as a correlate of local recombination rate in our study. Another factor that is related to GC content is the density of CpG methylation sites. DNA methylation has been suggested as a negative regulator of recombination [25]. Contrary to what could be predicted, the ratio of CpG to GpC, which represents an indicator of methylation intensity [25], was positively correlated to recombination rates. This finding suggests that methylation does not decrease local recombination rate in the honey bee at the scale that we investigated.

We did not find that single tri- or tetra-nucleotide motifs were more correlated with recombination than GC content or other, comparable tri- or tetra-nucleotide motifs. Particularly at the tetra-nucleotide level, many motifs exhibited very similar correlation coefficients, with GC content conspicuously related to the motif’s correlation to recombination. This relation was not strictly linear because tetra-nucleotide motifs with one A/T showed a stronger positive correlation to recombination rate than tetra-nucleotides that consisted solely of G/Cs. Similar results were obtained for di- and tri-nucleotides. This finding contrasts to studies at finer scales [25,26] that identified several specific motifs, including the tetra-nucleotide CGCA. The discrepancy may reflect differences in scale or statistical evaluation, or it may be due to biological differences. Overall, we conclude from our data sets that the correlation of a particular motif’s GC content to the overall GC content of the DNA may explain the motif’s correlation to recombination rate. This interpretation also fits most of the motifs identified in previous studies [25,26]. Any particular motif might provide specific binding sites for factors that facilitate chromatin access for the recombination initiation factors, resulting in recombination hotspots [22,37]. However, the honey bee genome seems to be devoid of distinct hotspots [25], corroborating our interpretation that no specific, cis-acting motifs control recombination rate in the honey bee genome.

Similar to a previous study of honey bee recombination at an intermediate scale [9], we found microsatellites, a particular form of simple repeat sequences, to be positively correlated to recombination rate. The abundance of microsatellites together with low complexity sequences may also influence the variance of local recombination rate in the bivariate analyses. Microsatellites have been mechanistically linked to recombination in-vitro [39], but our finding is the first that suggests that they may play a role in the evolutionary dynamics of a eukaryotic recombinational landscape. The high allelic diversity of microsatellites may cause intra-specific variation in local DNA structure that in turn could influence the frequency of local recombination events. In addition, several of the short sequence motifs that were highly correlated with recombination in some but not in other maps also correlated with the variance of recombination rate. However, our study could not resolve the question whether this observed variation in recombination rate is directly linked to variation in frequency of these correlated motifs because we lack the specific genome sequence information from the individuals that gave rise to our mapping populations.

Thus, we cannot rule out that evolution of specific binding sites, such as those for the zinc-finger protein PRDM9 [24], is causing some of the variation and more comparative analyses at a fine scale (e.g. [7]) will be needed in the future. In our current study, at least a part of the observed variation in recombination rate was due to the different spatial resolution that the eight linkage maps provide. However, we also observed considerable variability between pairs of maps that were matched in methodology and marker density. To date, surprisingly little is known about the variation of local recombination among genomes, although studies of this variability are important for understanding the evolution of recombination rates [27]. In yeast, local recombination rates were found relatively conserved even at a very fine scale despite overall differences in recombination [40] and no DNA features were identified to account for the remaining variation. In humans, variation in recombination hotspots have been linked to PRDM9 binding sites, but a similar mechanism could not be confirmed in *Drosophila*, probably due to multiple initiation processes for recombination [41]. In the highly recombining genome of the honey bee, multiple processes that lead to meiotic recombination also seem plausible, particularly with selection for high recombination counteracting the self-destruction of recognition motifs due to biased gene conversion [27].

## Conclusions

This study relied on eight recombination maps, which suggested a dynamic recombinational landscape of the honey bee genome, with a relatively low degree of conservation even at intermediate scales [29]. Our results robustly relate local GC content of the genome to the intra-genomic variation of recombination rates but not to the variability among maps. The importance of GC content suggests that DNA structure plays an important role in the frequent recombination of the honey bee genome but genome-wide fine-scale recombination maps and the precise mechanisms of the high recombination rate and the resulting local variability remain to be explored.

## Methods

Data from eight existing, genome-wide recombination maps of the honey bee were collected from different published studies. These maps were constructed in different laboratories using different genetic markers and marker densities. They showed variable levels of correlation with each other, with pairwise correlation coefficients calculated for 100 kbp windows ranging from 0.0 to 0.6 [29]. Based on 2000 microsatellite loci, the published “Solignac” map had the highest marker density, derived from a combination of two European families [11]. Two maps were based on approximately 1300 SNP markers genotyped with Illumina’s GoldenGate Assay™: the “Grooming” map [30] and the “VSH” map [31]. The “Grooming” map was generated from a backcross worker family in Mexico and the “VSH” map was derived from selected commercial stock in the USA. The “JH” map was based on a backcross between the divergently selected high and low pollen hoarding lines [42] and genotyped via RAD-tag sequencing, resulting in 1100 markers [43]. The remaining 4 maps each comprised about 230 evenly spaced SNP and microsatellite markers. Two of these crosses (“HBC” and “LBC”) were reciprocal backcrosses between the high and low pollen hoarding lines [44] and the other two (“R3” and “R5”) were two parallel backcrosses between a hybrid queen derived from the Africanized and European honey bee populations in North America and an Africanized male [45].

**Figure 3:**
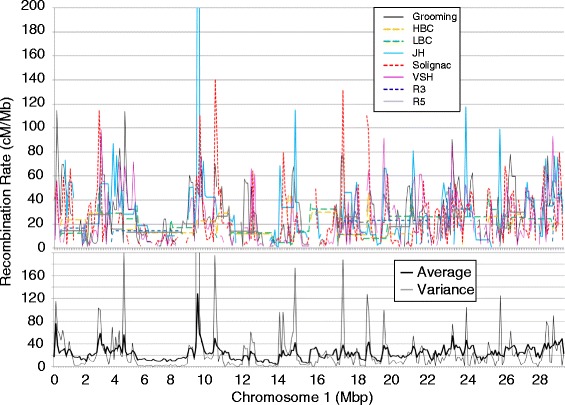
**The local recombination rates of eight diverse recombination maps were analysed individually (above), along with their average and variance (below), computed in 100 kb windows.** Data for the first chromosome are shown here as an example of the recombination data; all other chromosomes are depicted in the Additional file 1.

For all genetic markers of the eight maps the physical position in the current version of the honey bee genome (Amel 4.5) was determined by BLAST-n searches [46]. Maps were surveyed for inconsistencies between the genetic and the physical position of markers. Inter-marker intervals that were defined by a marker with conflicting genetic and physical locations were excluded from the subsequent analyses. The genome was divided into 100 kbp windows as the basic analysis unit. The recombination rate per window was computed as the weighted average of the corresponding inter-marker intervals. Intervals with a recombination rate equal to zero were excluded from the analysis because these values were artifacts, usually resulting from the combination of missing data and a pair of physically very close markers that did not experience any recombination. In addition to the local recombination rate estimates of each map, an overall average recombination rate and the variance of recombination rate among the eight maps were calculated for each window (e.g. chromosome 1 in Figure 3; all other chromosomes: see Additional file 1).

The DNA sequence and annotation features were downloaded from the NCBI website (ftp://ftp.ncbi.nih.gov/genomes/Apis_mellifera/Assembled_chromosomes/seq/) and local sequence features and gene annotations were extracted with their positional information. For each window, we computed GC content, CpG number, as well as a few select longer motifs [26] and the abundance of low complexity and all microsatellite sequences. The frequency of all di-, tri-, and tetra-nucleotide motifs per window were also determined by simple string searches. Even though these simple motifs presumably do not represent entire protein binding sites, analyzing these relatively short motifs allowed the evaluation of the complete parameter space (all 16, 64, and 256 possible combinations of bases, respectively) in a reasonable computational time. These motifs are partially contained in each other or constitute reverse complements of each other. Nevertheless, we tested each possible motif separately to provide a complete and unbiased analysis. While any true sequence motif that influences recombination by protein binding is likely longer, a specific subset of the shorter motifs should be included in the longer motif and hence show a strong correlation with recombination. In addition, the number and size of genes, their average distance, and the average size of exons according to the latest genome annotation [21] were compiled.

Subsequent analyses were performed for each genetic map independently, as well as for the average and variance of local recombination rates across maps. The relation between recombination rates and DNA sequence features was analyzed by calculating all bivariate correlation coefficients, followed by a stepwise backwards regression model (exclusion threshold: p ≥ 0.05). The di-, tri-, and tetra-nucleotide motifs were each analyzed in separate procedures due to their high correlation with each other and with some of the other DNA features. Again, bivariate correlation coefficients were computed. However, the high degree of collinearity required a principal component analysis (PCA) before relating the extracted principal components (PC) to recombination rates.

## Additional file

Additional file 1:
**Figures of the calculated local recombination rates along chromosomes 2 – 16 from eight different linkage maps, along with the average and variance of recombination rates, display the considerable heterogeneity of local recombination in the honey bee genome.**
