# Supplementary figures and images for: Genomic correlates of recombination rate and its variability across eight recombination maps in the western honey bee (Apis mellifera L.)
Source: BMC Genomics. 2015 Feb 21;16(1):107. doi: 10.1186/s12864-015-1281-2 (PMC4339005; doi:10.1186/s12864-015-1281-2)

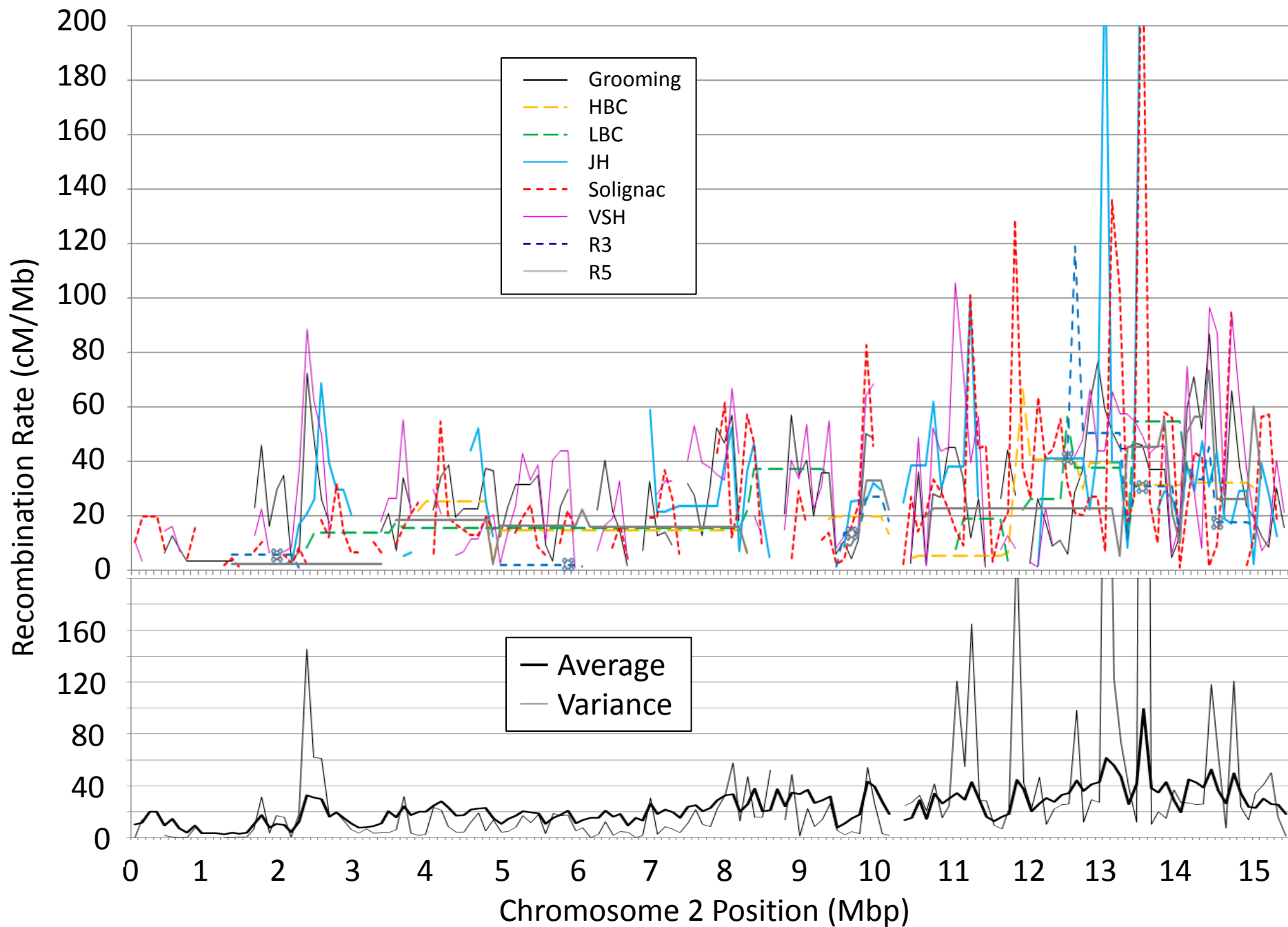

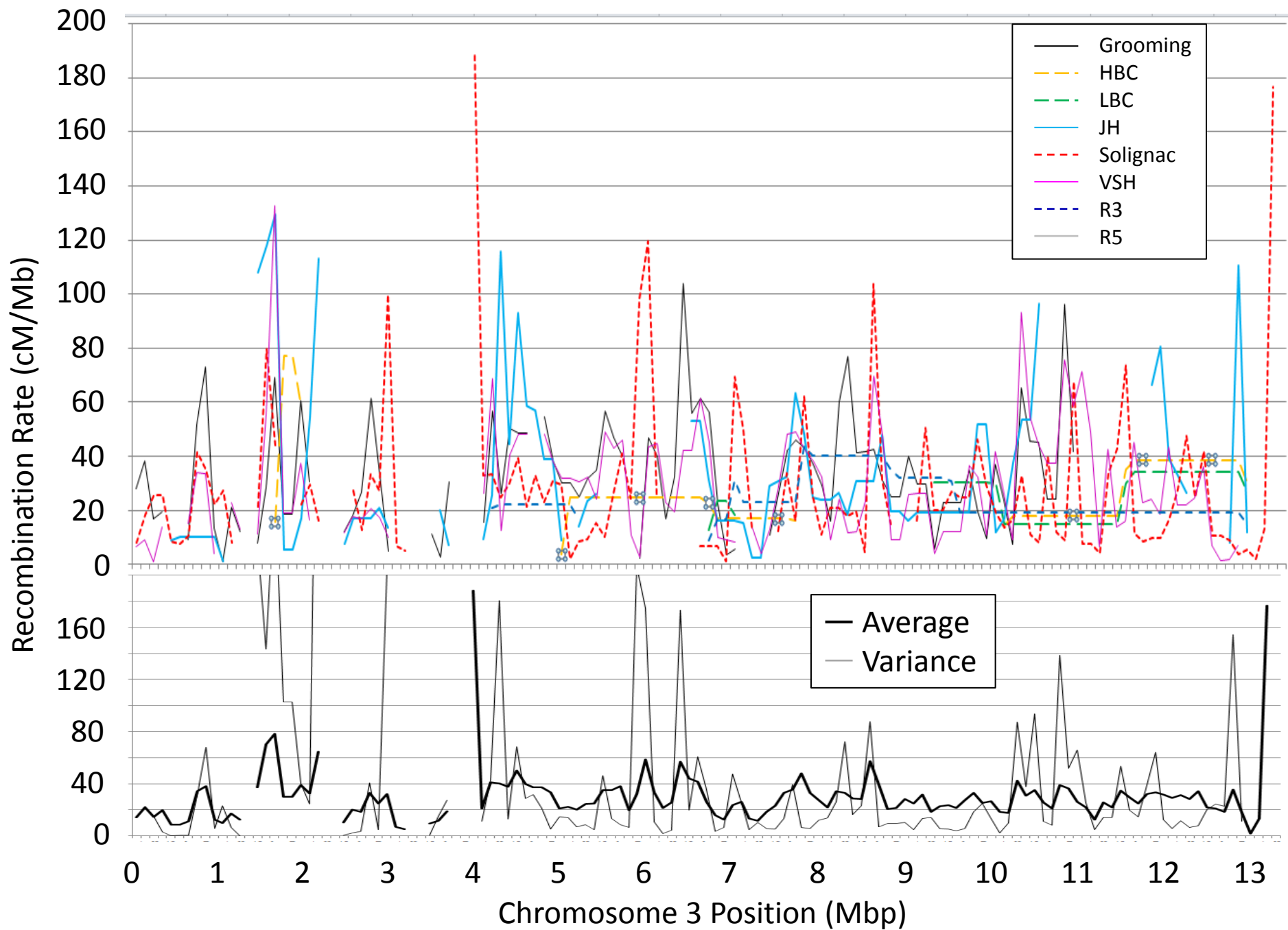

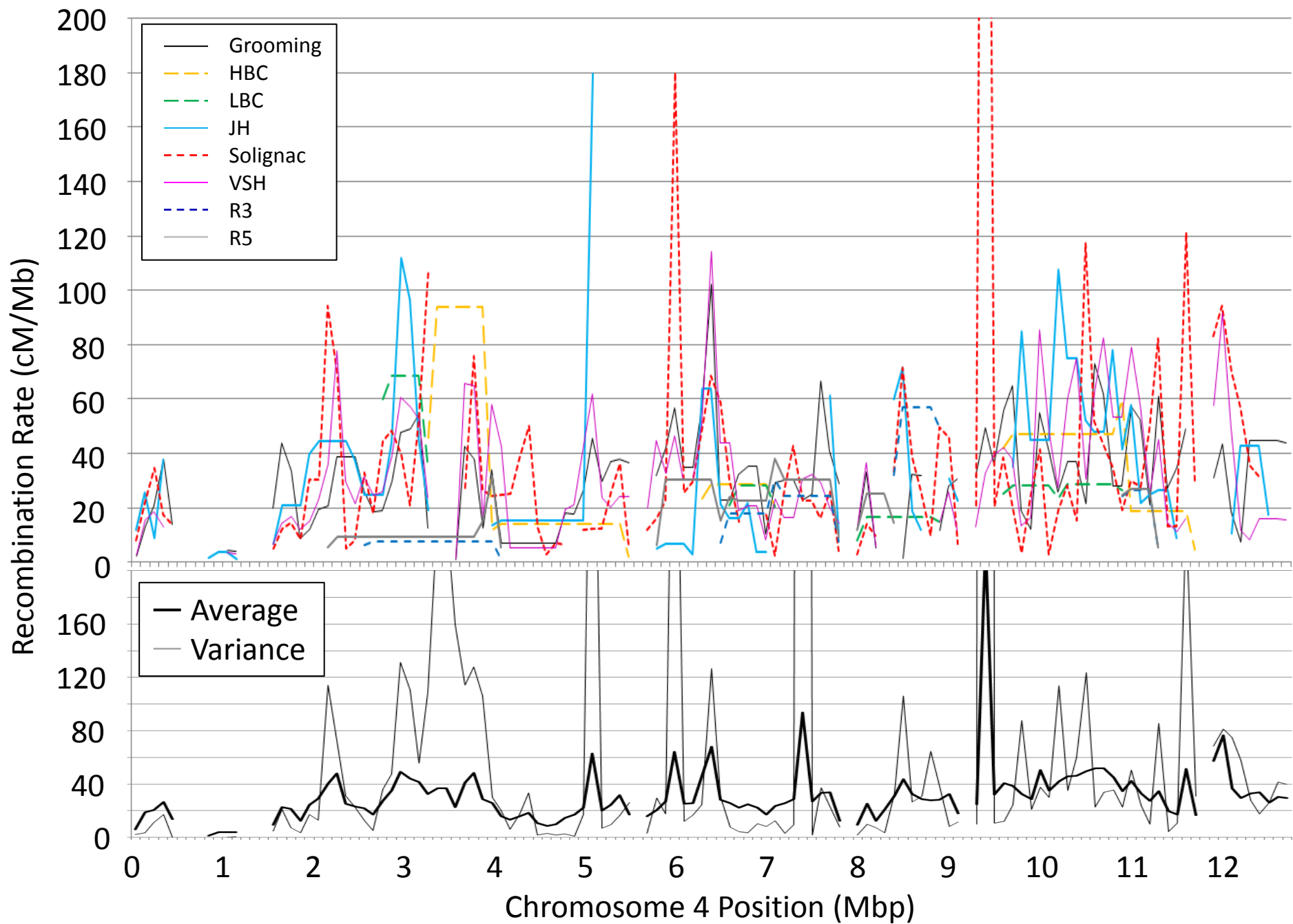

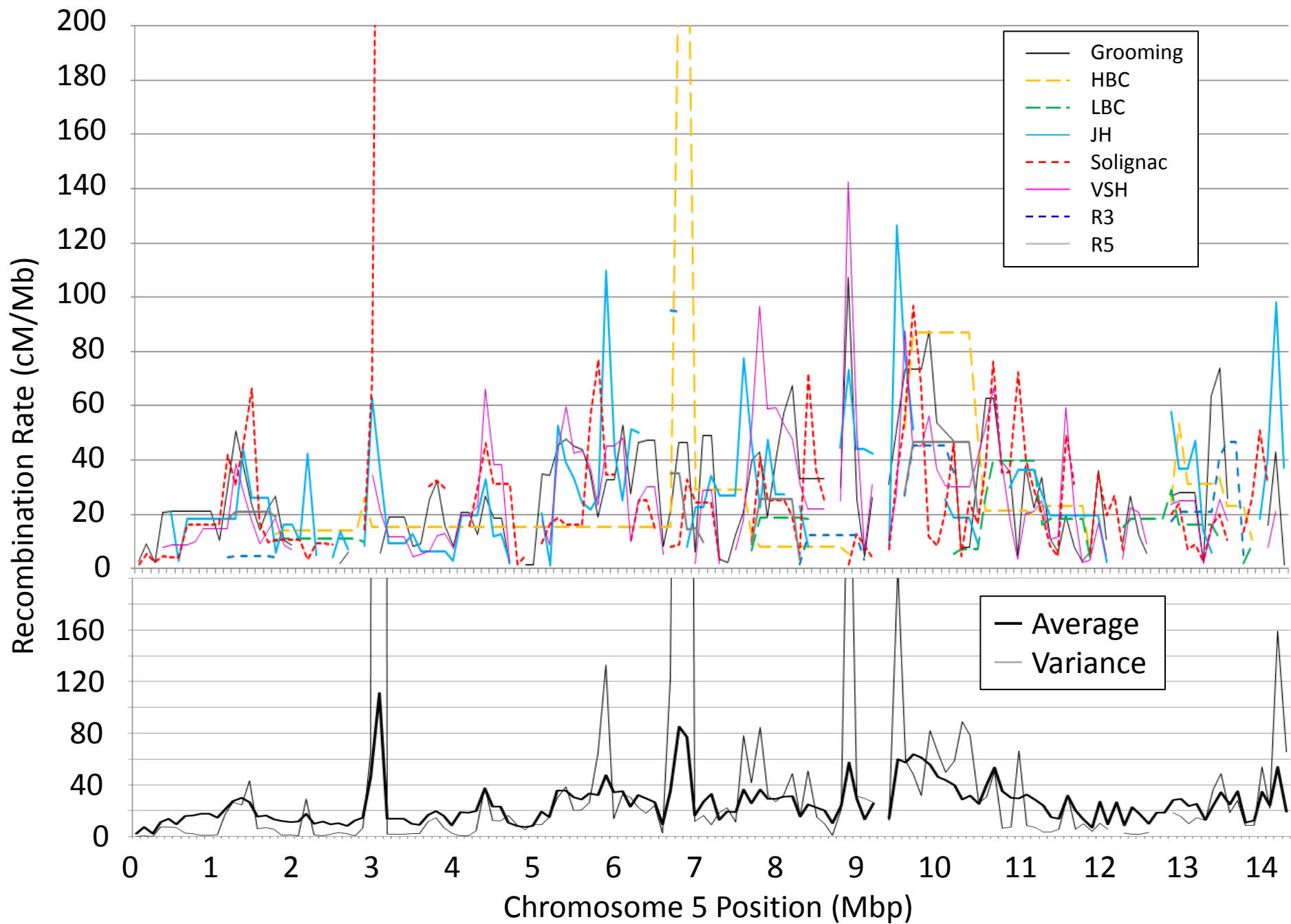

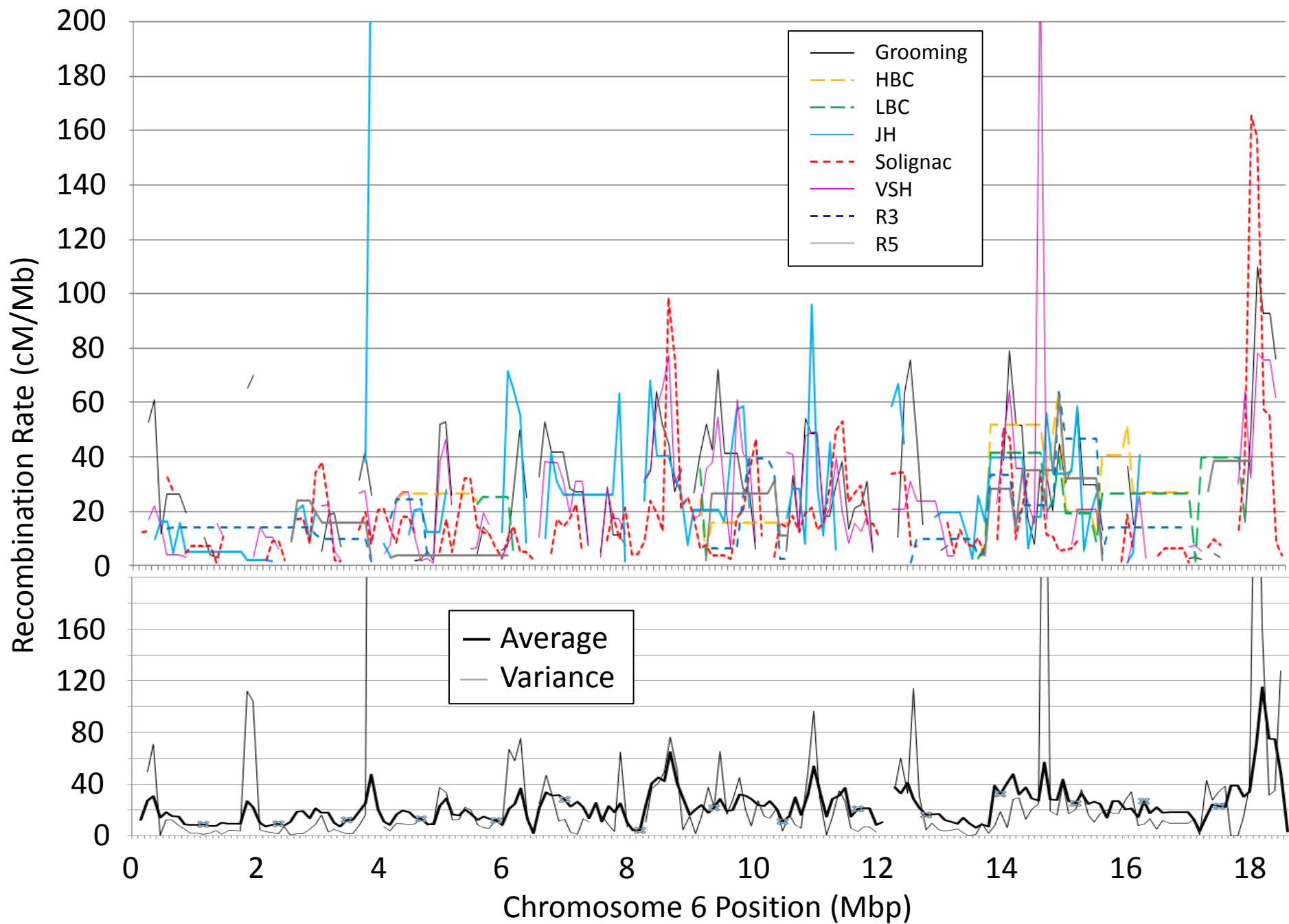

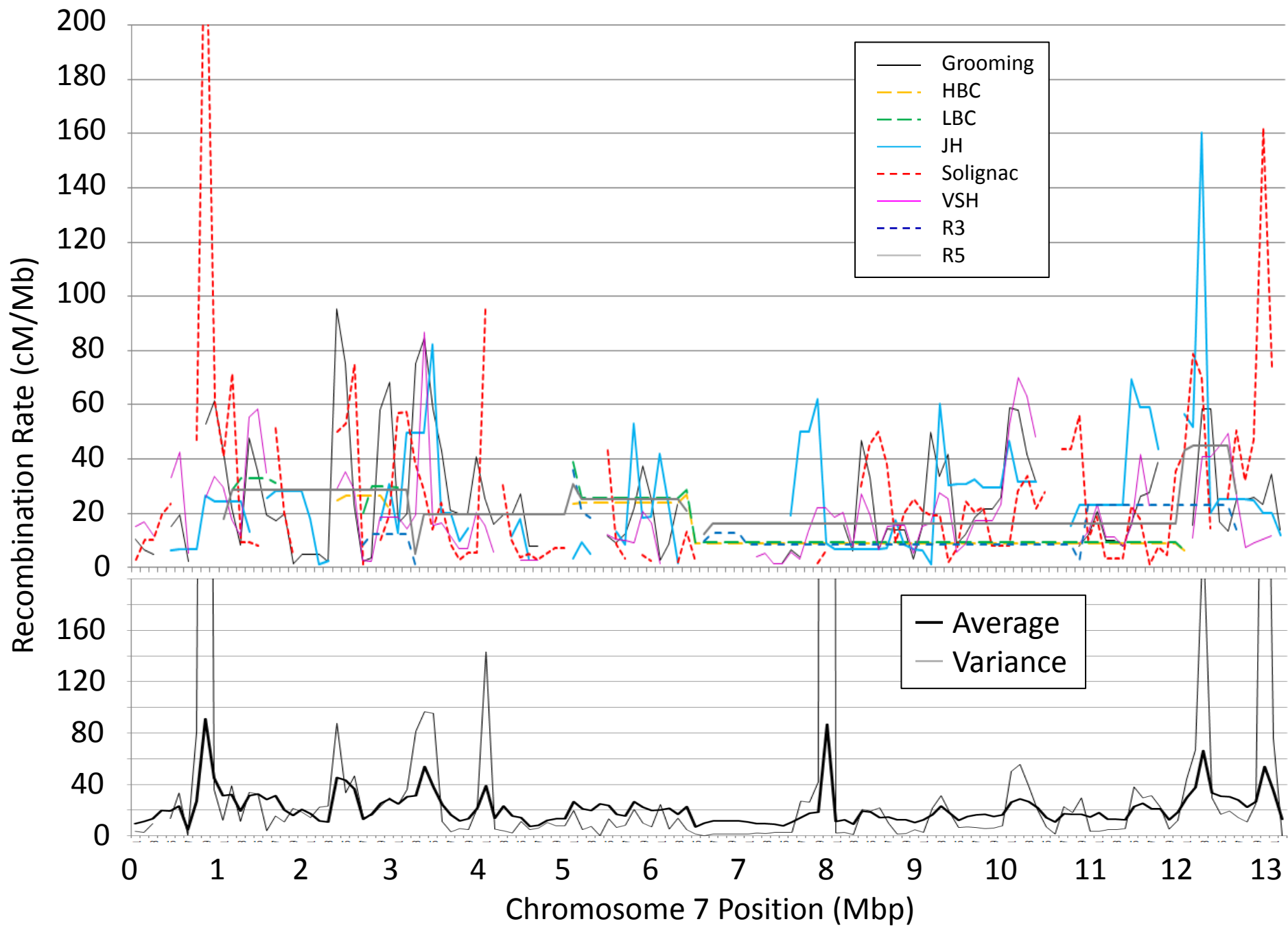

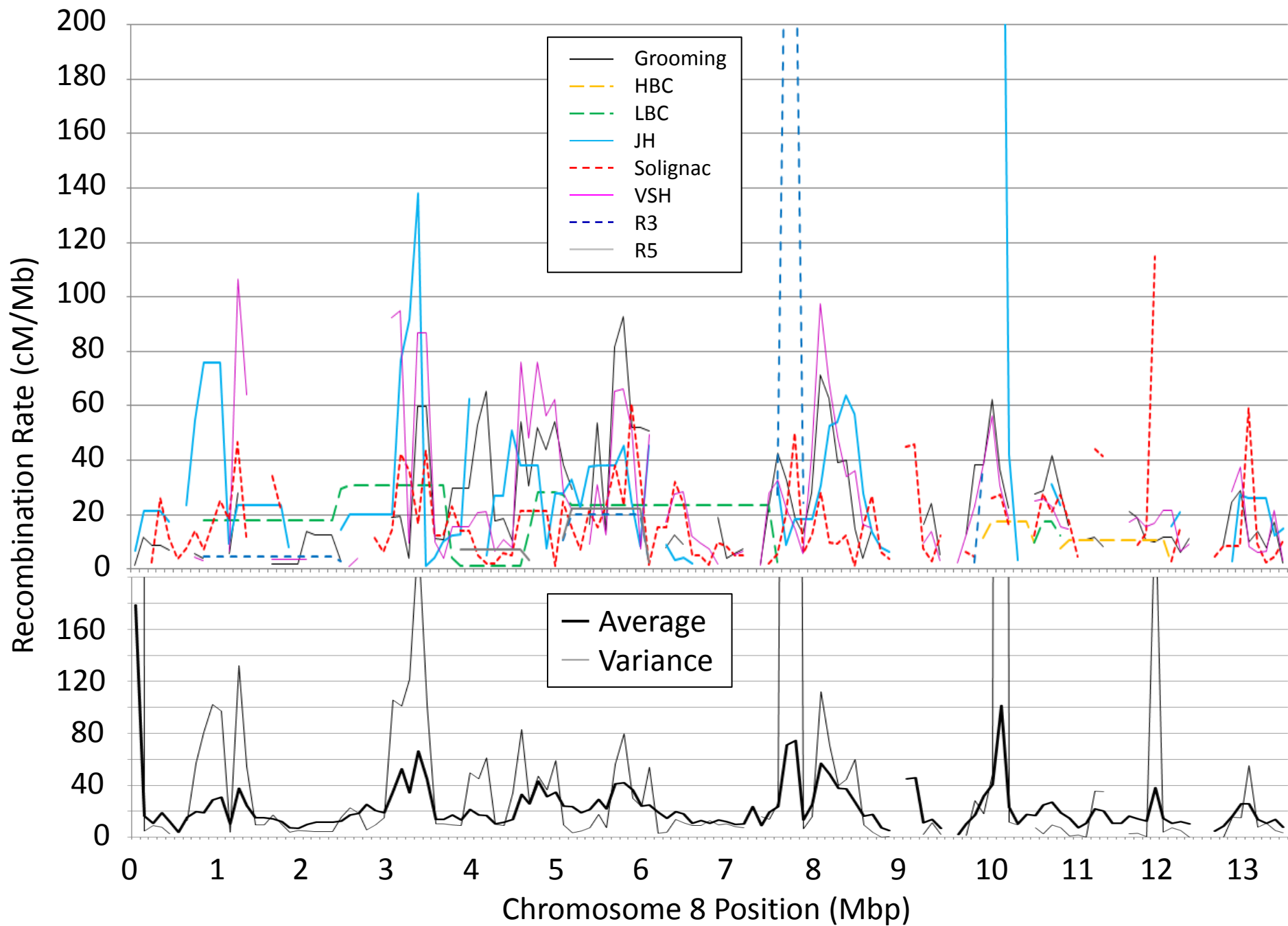

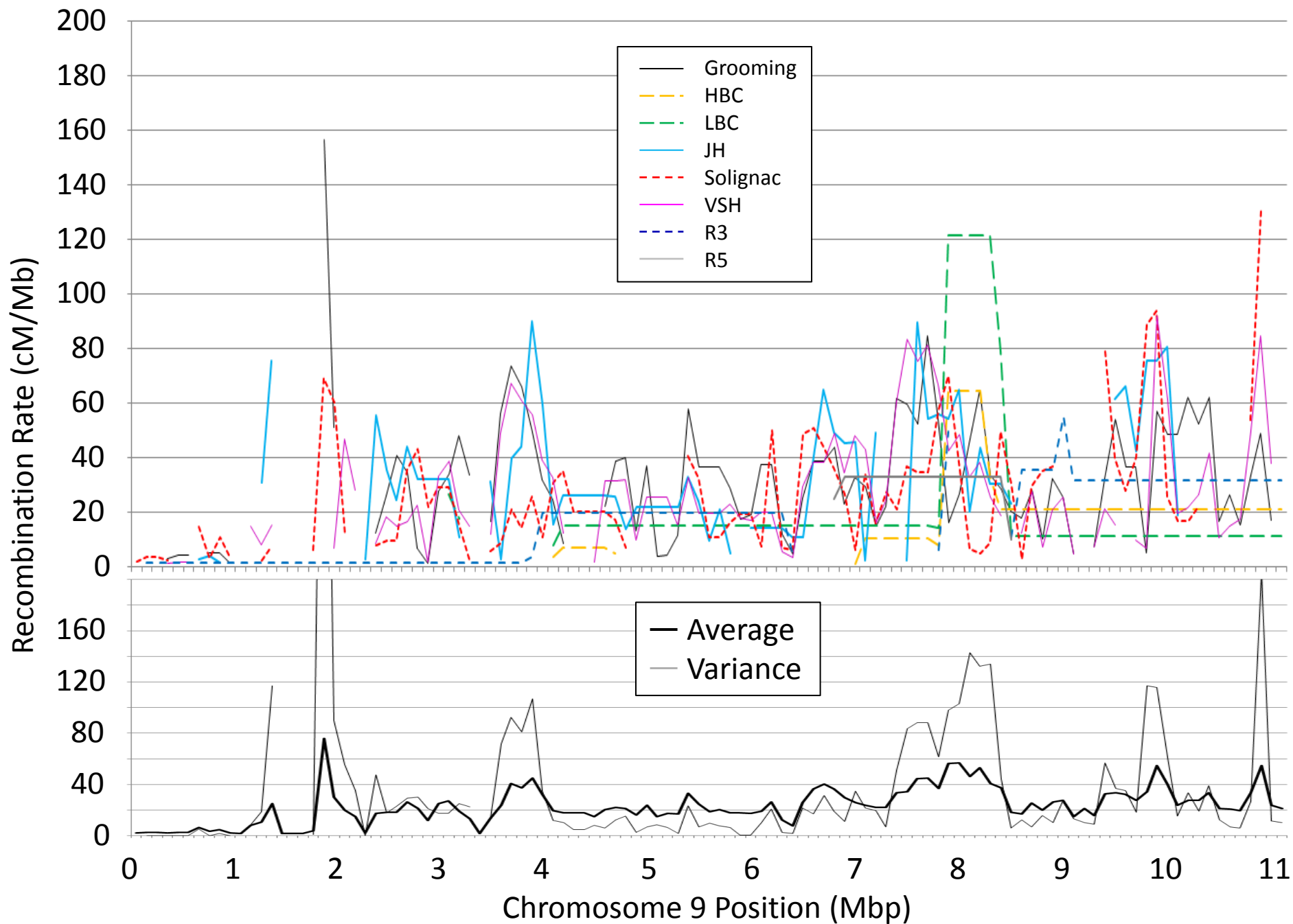

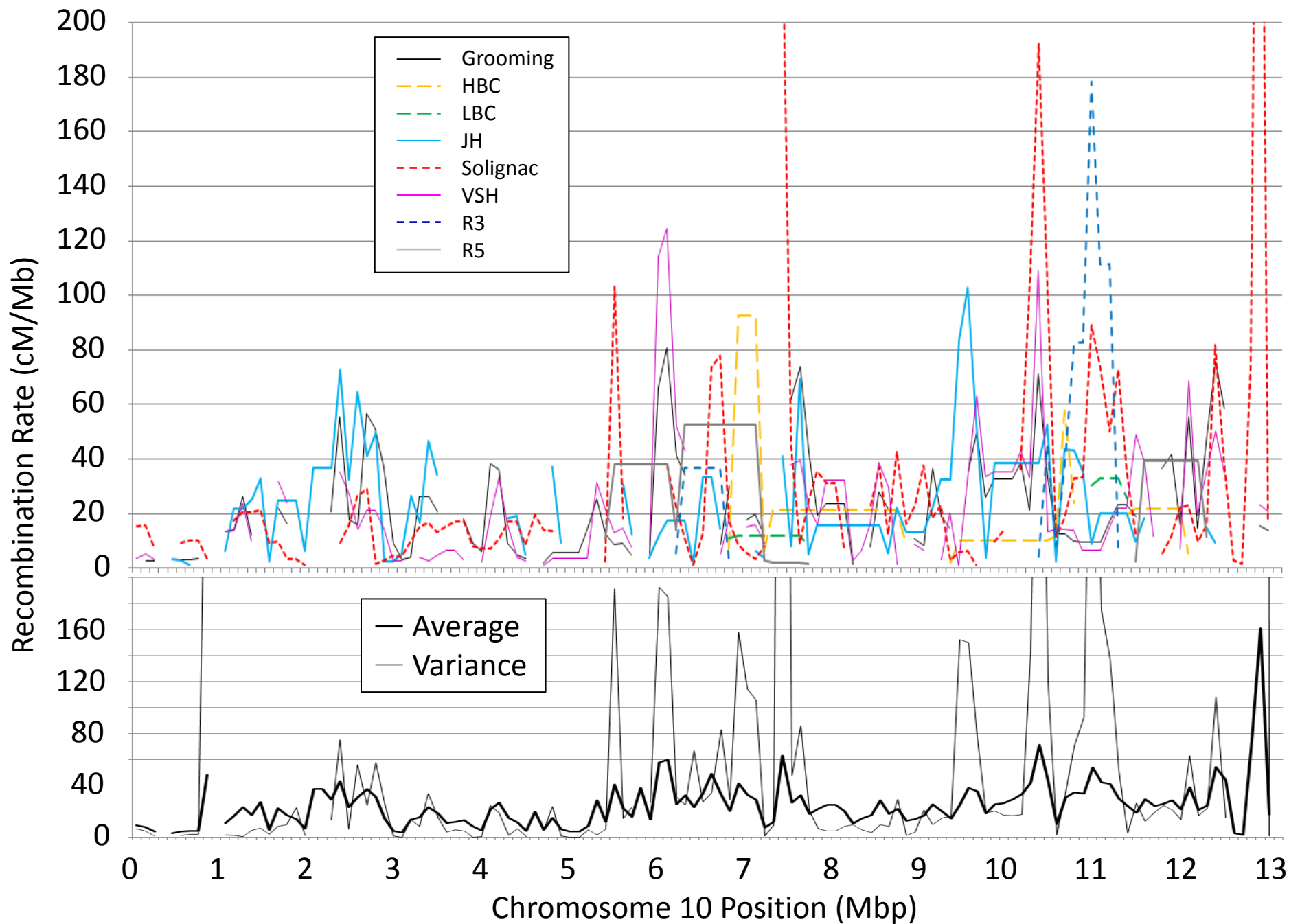

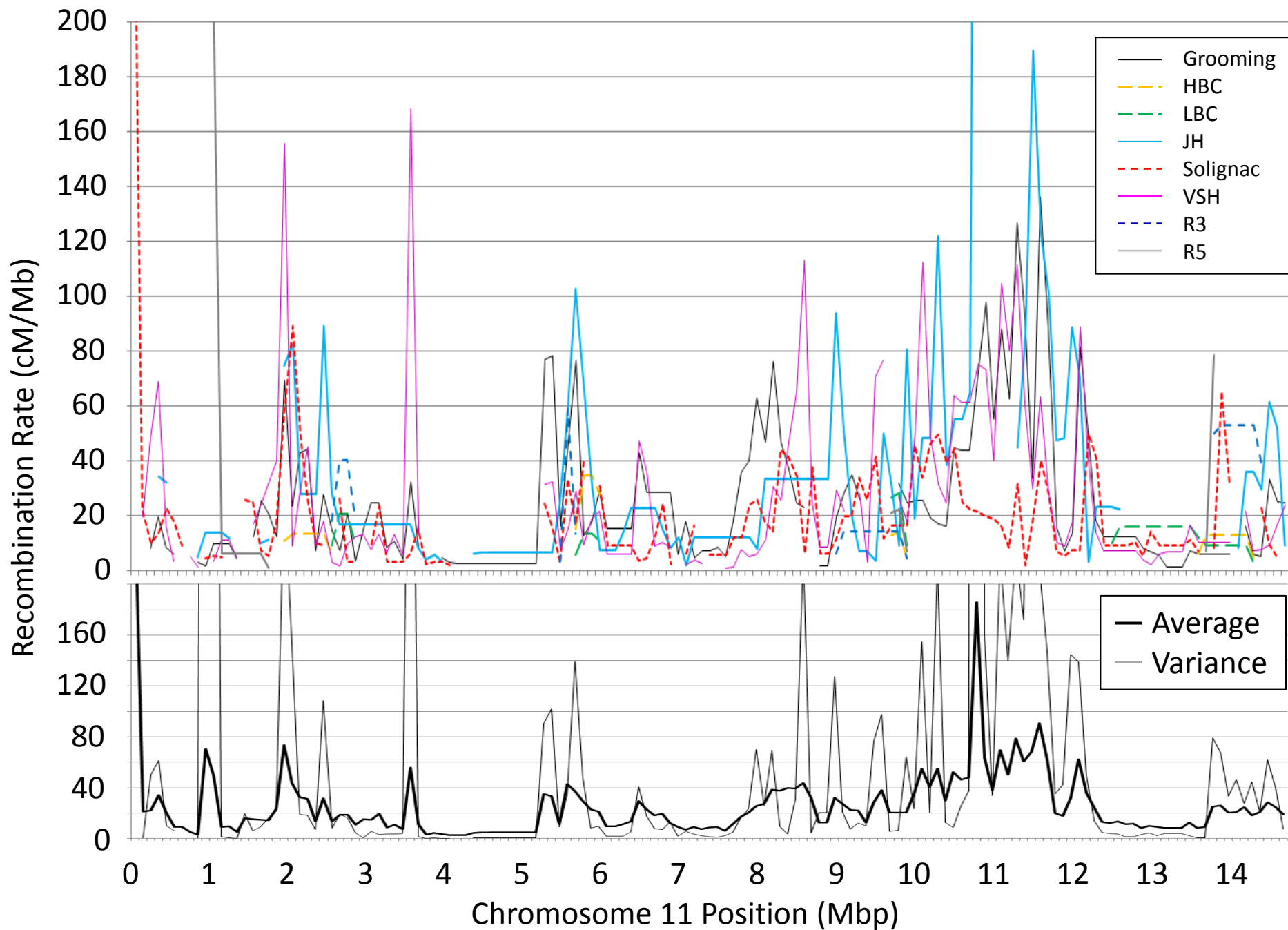

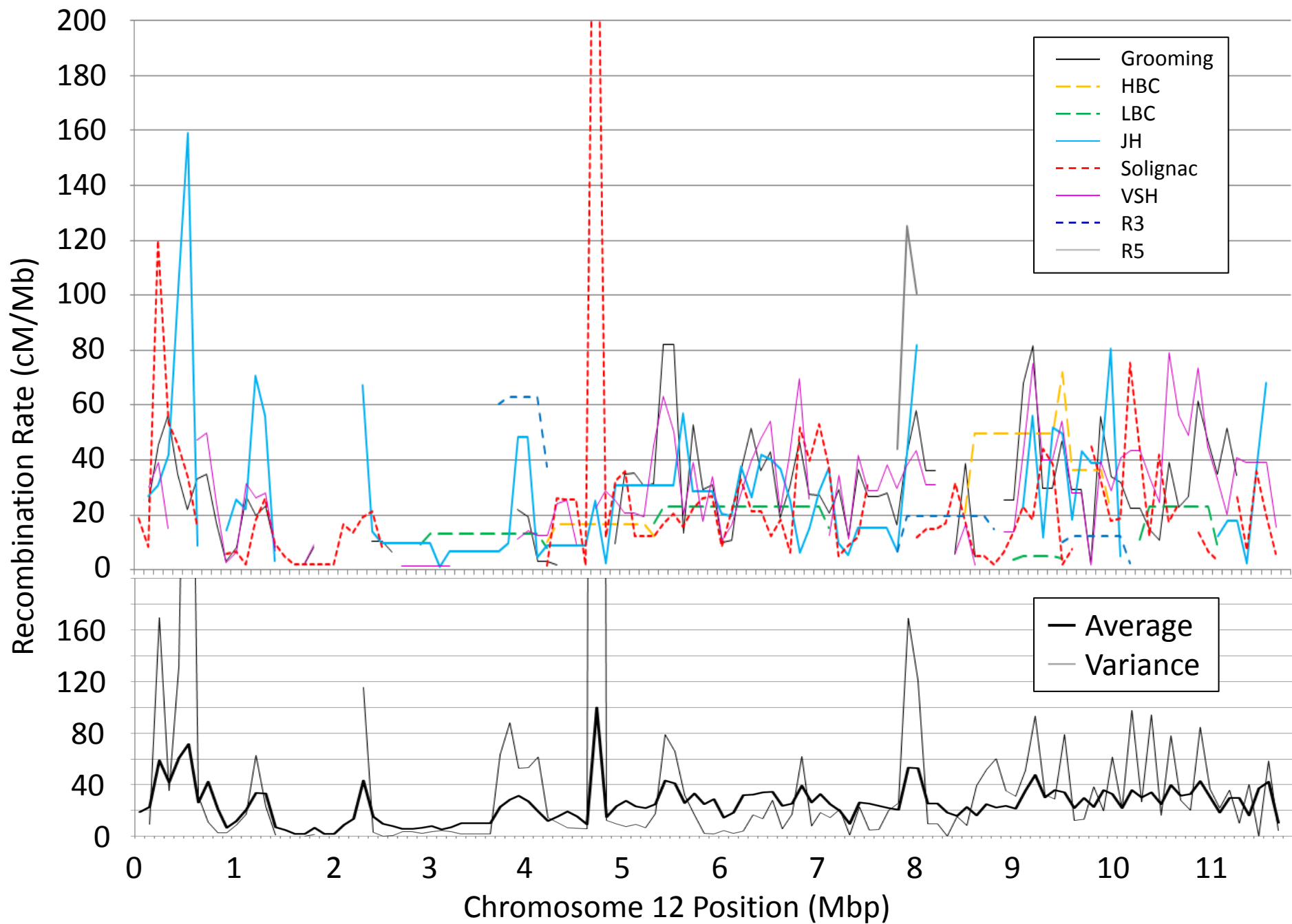

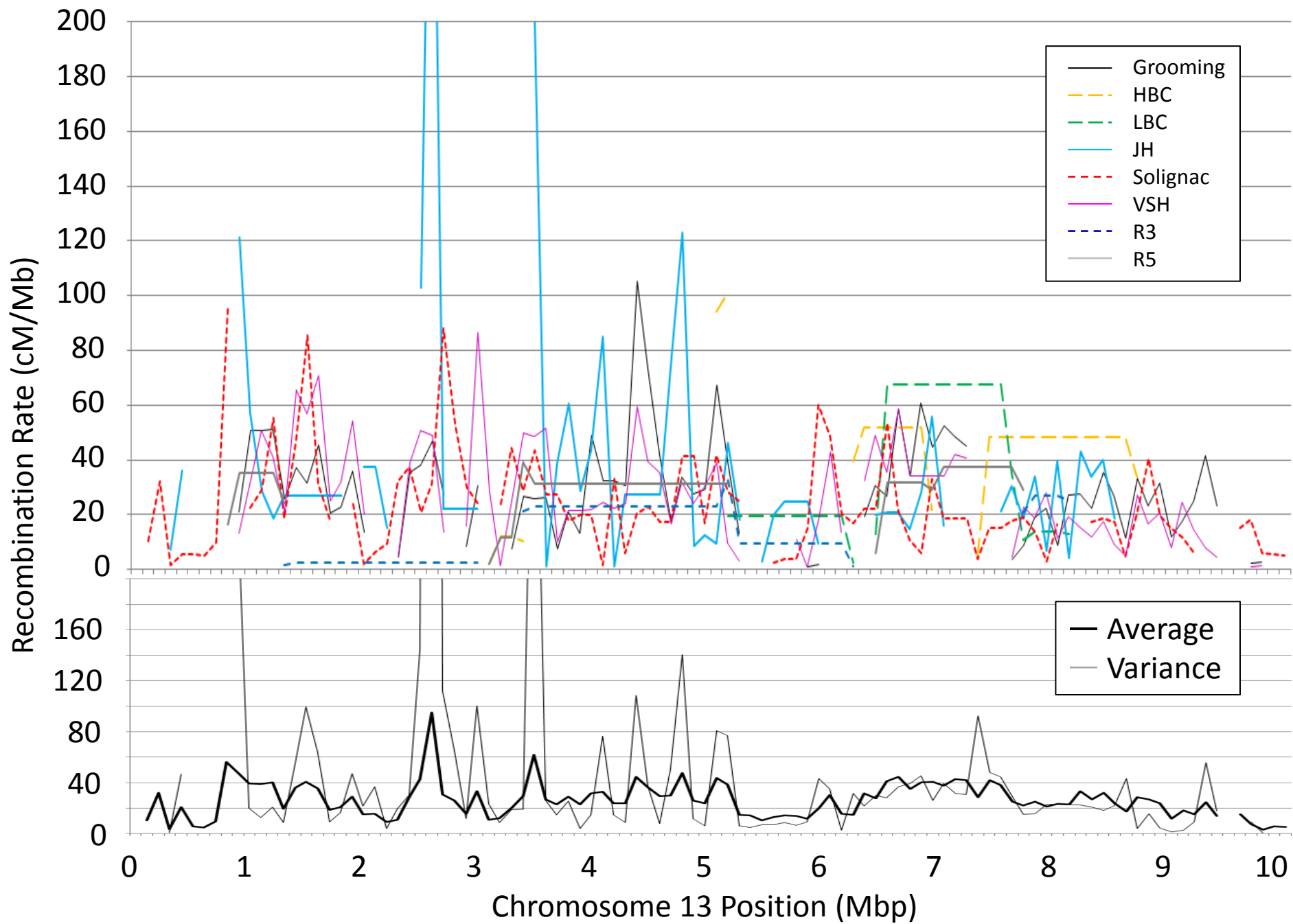

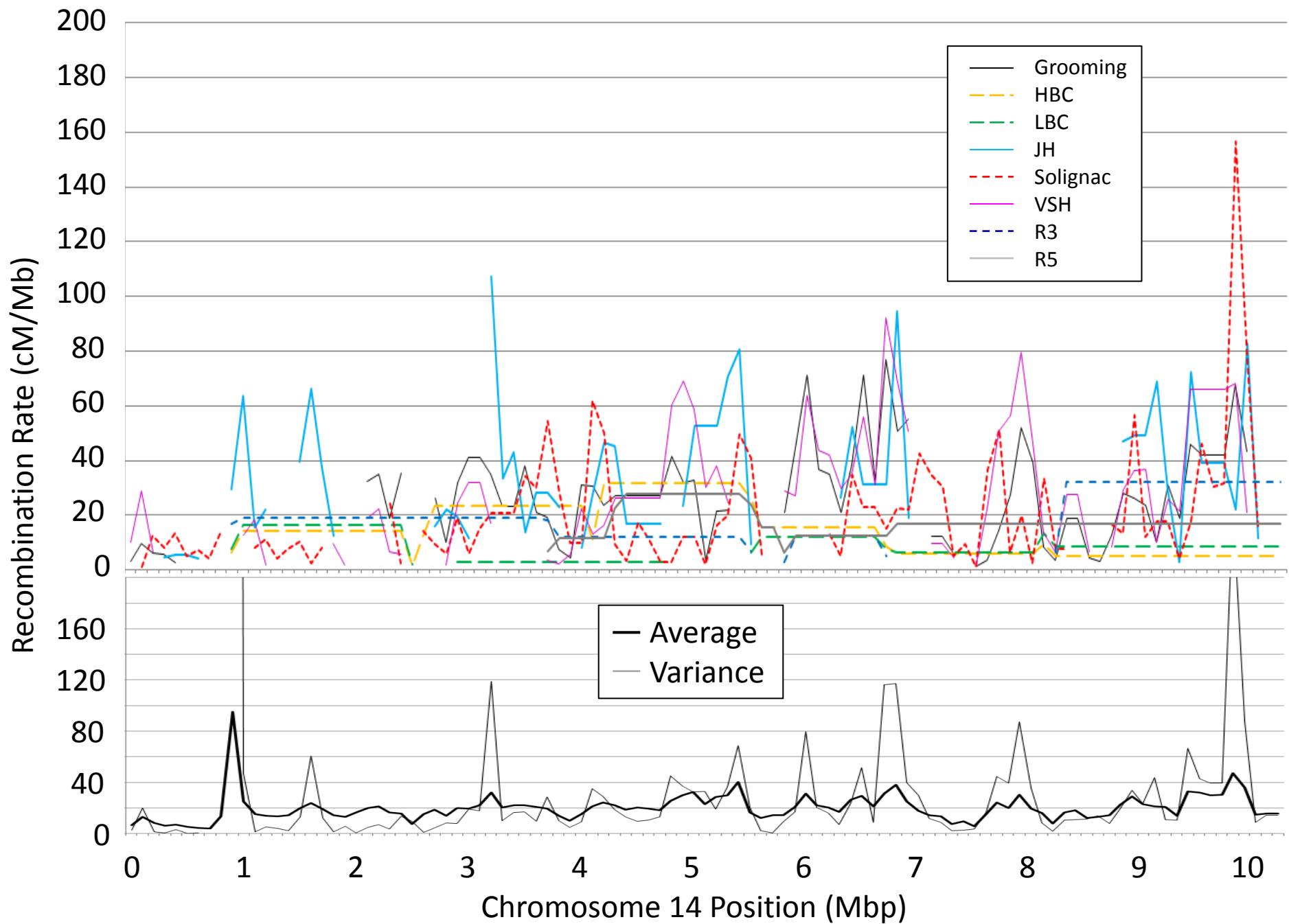

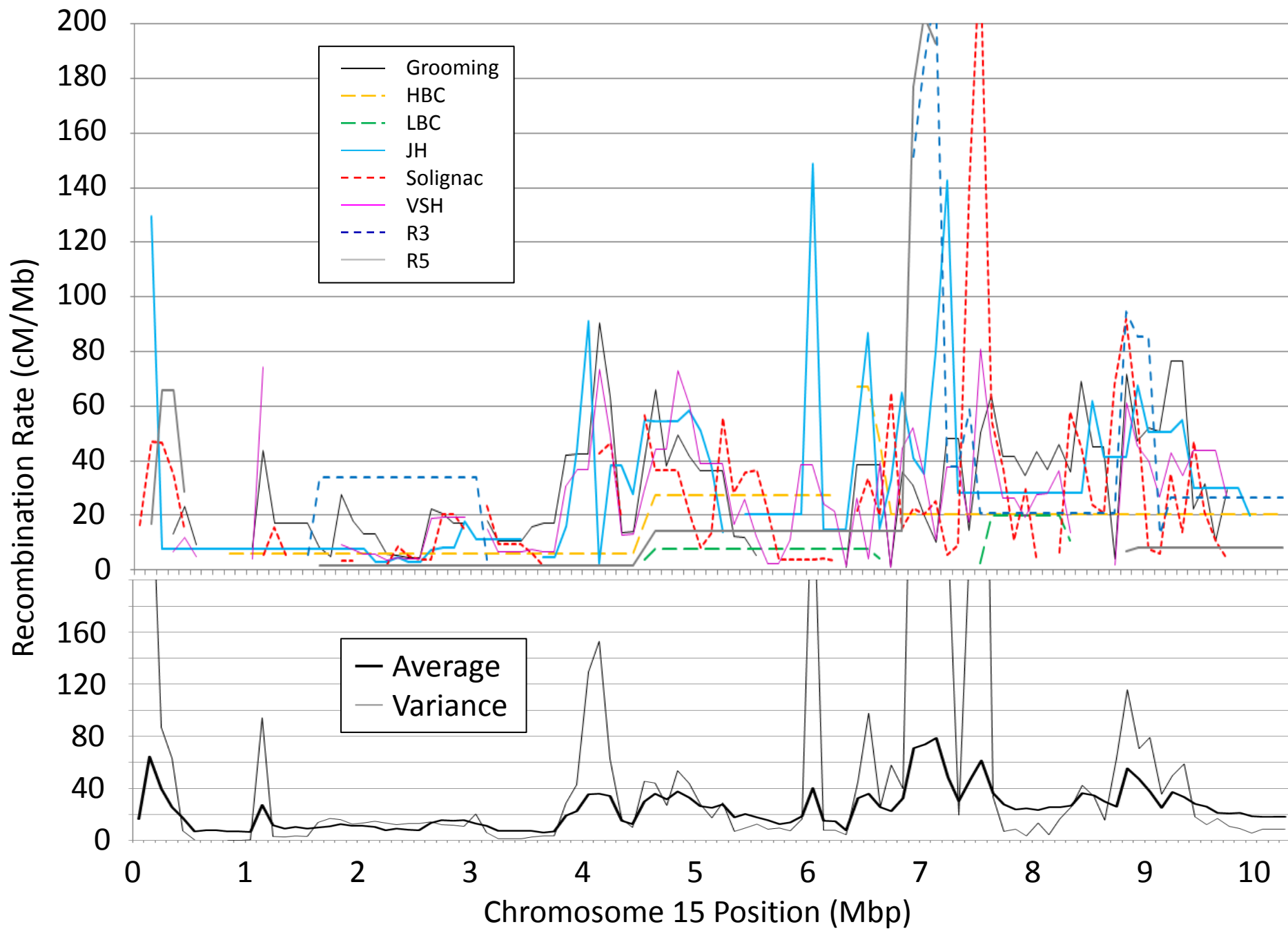

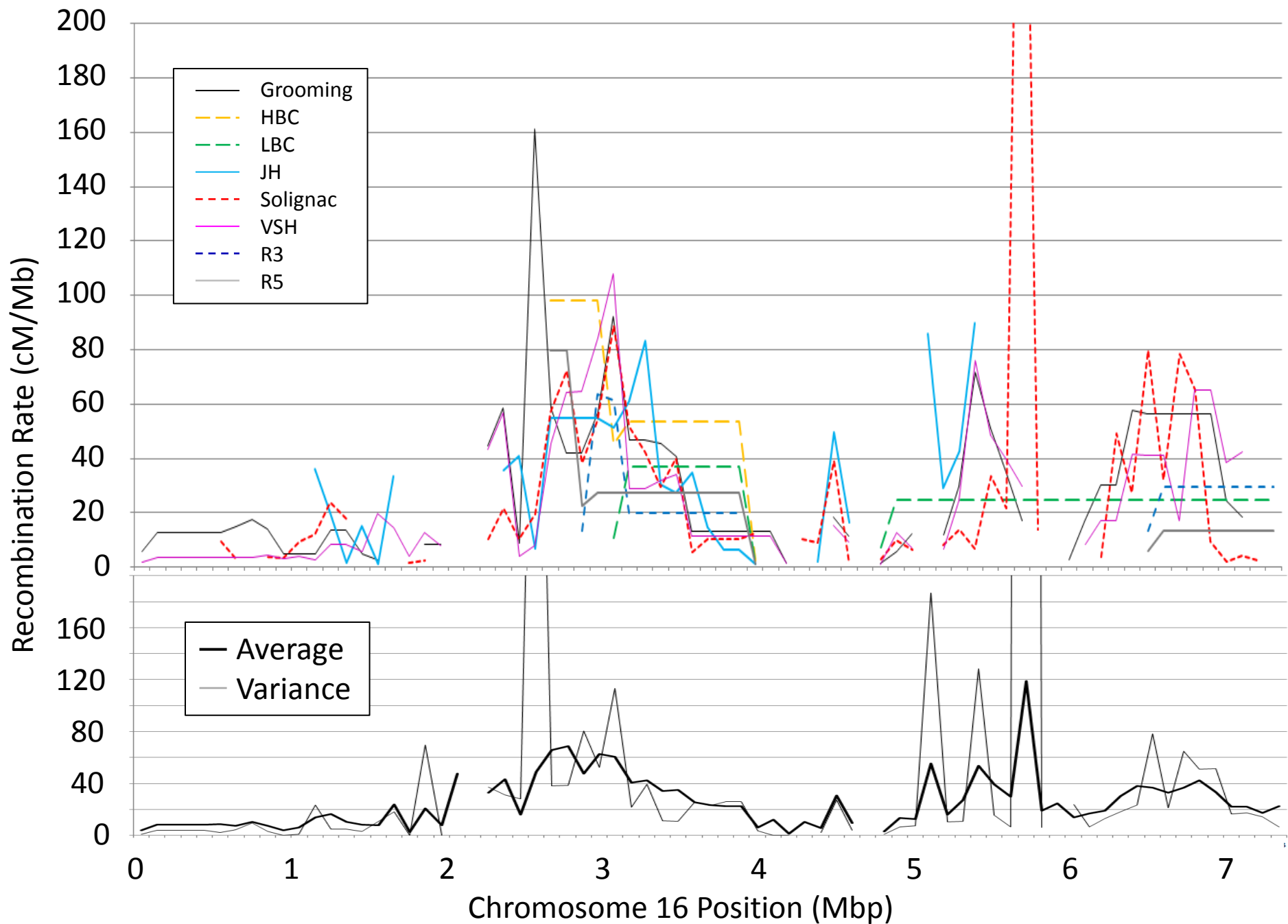

Supplement: Additional file 1: — Figures of the calculated local recombination rates along chromosomes 2 – 16 from eight different linkage maps, along with the average and variance of recombination rates, display the considerable heterogeneity of local recombination in the honey bee genome. [file 12864_2015_1281_MOESM1_ESM.pdf]
